# Impact of the COVID-19 pandemic in 2020 on the diagnosis, treatment, and prognosis of major cancers

**DOI:** 10.1097/JS9.0000000000003266

**Published:** 2025-08-26

**Authors:** Yaxiong Tang, Lede Lin, Zeyu Han, Xianyanling Yi, Xuanji Li, Linghao Meng, Ye Zhu, Jianzhong Ai

**Affiliations:** a Department of Urology, West China Hospital, Sichuan University, Chengdu, China; b Department of Cardiology, West China Hospital, Sichuan University, Chengdu, China

**Keywords:** cancers, cancer-specific survival, COVID-19, diagnosis, treatment

## Abstract

**Background:**

The global COVID-19 pandemic in 2020 has exerted multidimensional effects on cancer, yet detailed investigations into these impacts remain largely insufficient.

**Patients and methods:**

We evaluated the impact of the COVID-19 pandemic in 2020 on cancers of six major sites, including lung & bronchus, female breast, prostate, colon & rectum, pancreas, and corpus & uterus. Annual changes in the number of new cases were assessed using the Surveillance, Epidemiology, and End Results (SEER)-22 dataset. The impact of the epidemic on diagnosis, treatment, and prognosis was analyzed using the SEER-17 dataset. Specifically, Ordinal logistic regression models were used to investigate the impact of the pandemic on stage. Logistic regression was used to elucidate the impact of the pandemic on diagnosis in different subgroups. Finally, we assessed both the time interval from diagnosis to treatment and cancer-specific survival (CSS).

**Results:**

Compared with 2019, new diagnoses of the six major cancers decreased in 2020, with smaller changes in distant-stage cancers. Except for lung cancer (OR = 1.01, 95% CI, 0.97–1.05, *P* = 0.549), five of the six cancers diagnosed in 2020 tended to be at an advanced stage (all OR > 1, *P* < 0.005). During the pandemic, patients diagnosed with all six cancers in 2020 were more likely to have high-grade tumors, and for four cancers (excluding lung & bronchus and pancreatic cancers), patients were less likely to reside in areas with populations exceeding 1 million. No significant delay in the time from diagnosis to treatment was observed for any of the six cancers during the pandemic. With the exception of colorectal cancer (HR = 1.08, 95% CI, 1.02–1.14, *P* = 0.006), no significant deterioration in CSS was observed for the other five cancers (all *P* > 0.05).

**Conclusion:**

The number of new cases of the six major cancers decreased during the COVID-19 pandemic, but the impact on patients with different characteristics was different. Most cancer types tended to be diagnosed at a later stage, but the timeliness of treatment was not adversely affected. Some cancer types exhibited significantly worse CSS, or a trend toward worse CSS, but longer follow-up is needed to confirm these findings.

## Introduction

The COVID-19 pandemic in 2020 has exerted significant pressure on healthcare systems worldwide^[1–3]^. While lockdown-based containment measures, widely implemented by governments, achieved some success in controlling the spread of the virus, they also generated substantial negative effects on the manage-ment of other diseases. For example, these measures led to delayed presentations and reduced surgeries among myocardial infarction patients, as well as decreased HIV testing rates^[4–6]^. The COVID-19 pandemic in 2020 has also imposed multidimensional challenges on cancer management^[7–9]^. Previous studies have widely reported that, during the pandemic, reductions in cancer screening^[10,11]^, delays or decreases in cancer diagnoses^[9,12,13]^, and interruptions in cancer treatment were commonly observed^[8,14,15]^. These disruptions may ultimately contribute to a deterioration in cancer prognosis^[16]^. Despite these findings, most existing studies have concentrated on a single cancer type or a specific aspect of impact, lacking a comprehensive evaluation across multiple dimensions. The relatively short study periods have also made it challenging to capture the potential long-term effects of the pandemic on prognosis. Furthermore, many studies are based on data from single clinical centers, which limits the ability to comprehensively reflect the true situation across the broader population.

**HIGHLIGHTS**

- During the COVID-19 pandemic in 2020, new diagnoses of six major cancers (lung cancer, female breast cancer, prostate cancer, colorectal cancer, pancreatic cancer, and corpus & uterus cancer) decreased compared to 2019, with most cancers tending to be diagnosed at a later stage, except for lung cancer.
- Despite the pandemic, no significant delays were observed in the time interval from cancer diagnosis to treatment for any of the six major cancers.
- Some cancer types, including prostate, colorectal, and corpus & uterus cancers, exhibited significant cancer-specific survival (CSS) declines or trends toward worse outcomes; however, longer follow-up is needed to confirm these findings.

In this study, we used data from the Surveillance, Epidemiology, and End Results (SEER) database. Our aim was to comprehensively investigate the multidimensional effects of the COVID-19 pandemic in 2020 on six major cancers, including lung cancer, female breast cancer, prostate cancer, colorectal cancer, pancreatic cancer, and corpus & uterus cancer. Specifically, we analyze the pandemic’s impact on the number of diagnosed cases, stage at diagnosis, patient demographics, time intervals from diagnosis to treatment, and prognosis, with the aim of deriving insights from population-based data analysis. This study has been reported in line with the STROCSS guidelines^[17]^.

## Patients and methods

### Data source and patient selection

The SEER database, from the National Cancer Institute, covers about 50% of the U.S. population and is widely used in cancer epidemiology research^[18]^. In this study, we utilized the SEER-17 dataset and SEER-22 dataset, which cover 26.5% and 47.9% of the U.S. population, respectively^[19,20]^. Since the data were anonymized, ethical review by our institution and informed consent from the patients were exempted. This study complies with the Declaration of Helsinki and is recorded in the Research Registry. Cancers of six anatomical sites, including lung & bronchus, female breast, prostate, colon & rectum, pancreas, and corpus & uterus, were identified using the International Classification of Diseases for Oncology, 3rd Edition (ICD-O-3) codes.

The SEER-22 dataset, which cover a wider population (47.9% of the U.S. population), were used to clarify the impact of the COVID-19 pandemic in 2020 on the number of new cases. All patients aged 20–85 years with cancer at the above six sites diagnosed between 2017 and 2021 were included in the SEER-22 dataset^[20]^. The SEER-17 dataset provides more comprehensive demographic and prognostic information. Thus, it was used to further analyze the impact of the COVID-19 pandemic in 2020 on diagnosis, treatment, and prognosis for these six cancers^[19]^. Patients aged 20–85 years with a single primary cancer at the six sites mentioned above diagnosed between 2019 and 2020 were included, while those with incomplete information on demographic variables of interest (race, marital status, median household income, Rural-Urban continuum), cancer stage, time from diagnosis to treatment, and follow-up information (cause of death and follow-up time) were excluded. Excluded patients accounted for less than 10% of the overall population.

### Statistical analysis

Based on the SEER-22 dataset, we first described the changes in the number of new cases of six major cancers diagnosed between 2017 and 2021. It is well known that the Centers for Disease Control and Prevention (CDC) announced the first case of severe acute respiratory syndrome coronavirus 2 (SARS-CoV-2) infection on 20 January 2020, marking the beginning of the COVID-19 pandemic in the United States^[1–3]^. Therefore, we focused on the changes in newly diagnosed cases in 2020 (the year of the COVID-19 pandemic) relative to 2019. Given that previous studies have shown that the impact of the pandemic on cancer incidence varies by stage, we further analyzed the changes in the number of new cases by cancer stage^[9]^.

Based on the SEER-17 dataset, we then clarified the impact of the COVID-19 pandemic in 2020 on the diagnosis, treatment, and prognosis of six major cancers. Patients were categorized into two groups based on year of diagnosis: those diagnosed in 2020 were assigned to the COVID-19 impact group, while those diagnosed in 2019 served as the control group. Baseline differences between the groups were compared using the Pearson chi-square test or the Wilcoxon rank sum test, as appropriate. To assess the impact of the COVID-19 pandemic in 2020 on the stage at cancer diagnosis, a multivariable ordered logistic regression model was employed. Cancer stage was treated as an ordered outcome variable (Distant > Regional > Localized), with the year of diagnosis as the primary explanatory variable. The model was adjusted for age, sex (for lung cancer, colorectal cancer, and pancreatic cancer), marital status, median household income, rural-urban continuum, pathological type, and tumor grade. For prostate cancer, prostate-specific antigen (PSA) level was also included as an additional covariate.

Further, a multivariable logistic regression was further performed to explore predictors of diagnosis in 2020 to clarify the changes in the characteristics of the diagnosed population during the COVID-19 pandemic. To assess the potential impact of the pandemic on treatment timeliness, the Wilcoxon rank sum test was employed to compare the time interval from diagnosis to treatment initiation between patients diagnosed in 2019 and 2020. Finally, Kaplan-Meier survival curves and the log-rank test were used to evaluate the effect of the COVID-19 pandemic in 2020 on cancer-specific survival (CSS) across the six cancers. All statistical analyses were conducted using R software (Version 4.4.2), with a two-sided *P*-value < 0.05 considered statistically significant.

### Reduced incidence of new cases during the COVID-19 pandemic

**Figure 1.**
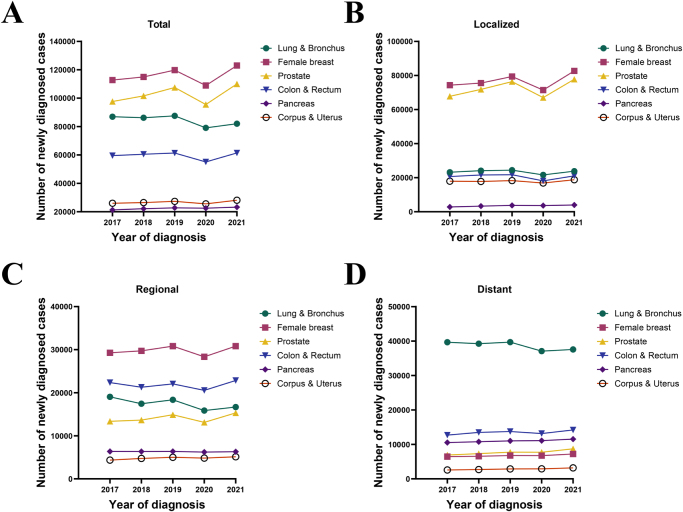
Total number of cases of the six major cancers diagnosed in the SEER-22 dataset from 2017 to 2021 (A), and the number of cases categorized by stage as localized (B), regional (C), and distant (D).

Figure 1 shows the changes in the number of new cases of six major cancers in the SEER-22 dataset from 2017 to 2021. From 2017 to 2019, the overall number of new cases of the six types of cancer increased annually, but the number of new cases in 2020 decreased significantly compared with 2019. Table 1 summarizes the overall and stage-specific changes in newly diagnosed cases in 2020 compared to 2019. Compared with 2019, the overall number of cases of lung cancer, female breast cancer, prostate cancer, colorectal cancer, pancreatic cancer, and corpus & uterus cancer diagnosed in 2020 decreased by 9.7%, 9.0%, 11.2%, 10.2%, 1.1%, and 6.5%, respectively. In terms of stage-specific trends, the number of new cases for patients with local and regional stages decreased in 2020 for all six cancers compared with 2019. For distant-stage lung cancer, female breast cancer, prostate cancer, and colorectal cancer, the number of new cases also declined in 2020, although the reduction was less pronounced than that observed for localized and regional stages. In contrast, the number of new cases of distant-stage pancreatic cancer and corpus & uterus cancer increased in 2020 by 0.8% and 1.2%, respectively, compared with 2019.

**Table 1:** The proportion of changes in the number of newly diagnosed cases of major cancers in 2020 compared with 2019, 22 SEER registries

Cancer sites	Cancer stage			
	Total	Localized	Regional	Distant
Lung & bronchus	−9.7%	−11.5%	−13.6%	−6.6%
Female breast	−9.0%	−10.0%	−8.0%	−0.4%
Prostate	−11.2%	−12.3%	−11.9%	−0.6%
Colon & rectum	−10.2%	−16.7%	−6.8%	−4.1%
Pancreas	−1.1%	−1.5%	−2.2%	0.8%
Corpus & uterus	−6.5%	−7.7%	−3.7%	1.2%

### Impact of the COVID-19 pandemic on cancer diagnosis

In the SEER-17 dataset, 40 998, 83 269, 55 682, 38 617, 10 913, and 20 580 patients with lung cancer, female breast cancer, prostate cancer, colorectal cancer, pancreatic cancer, and corpus & uterus cancer were included, respectively. Detailed baseline characteristics are provided in Supplemental Digital Content (Table S1, available at: http://links.lww.com/JS9/E937, Table S2, http://links.lww.com/JS9/E938, Table S3, http://links.lww.com/JS9/E939, Table S4, http://links.lww.com/JS9/E940, Table S5, http://links.lww.com/JS9/E941, Table S6, http://links.lww.com/JS9/E942).

**Figure 2.**
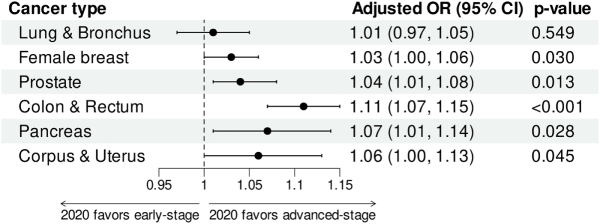
Multivariable ordinal logistic regression models assessed the impact of the COVID-19 pandemic in 2020 on cancer stage at diagnosis. Adjusted covariates included age, sex (for lung cancer, colorectal cancer, and pancreatic cancer), marital status, median household income, urban-rural continuum, pathology type, grade, and prostate-specific antigen for prostate cancer.

Figure 2 summarizes the effects of the COVID-19 pandemic in 2020 on stage at diagnosis. Patients diagnosed in 2020 were more likely to be diagnosed at a later stage compared to those diagnosed in 2019 for female breast cancer (OR = 1.03, 95% CI, 1.00–1.06, *P* = 0.030), prostate cancer (OR = 1.04, 95% CI, 1.01–1.08, *P* = 0.013), colorectal cancer (OR = 1.11, 95% CI, 1.07–1.15, *P* < 0.001), pancreatic cancer (OR = 1.07, 95% CI, 1.01–1.14, *P* = 0.028), and corpus & uterus cancer (OR = 1.06, 95% CI, 1.00–1.13, *P* = 0.045). However, the stage at diagnosis for lung cancer was not significantly affected by the year of diagnosis (OR = 1.01, 95% CI, 0.97–1.05, *P* = 0.549).

Supplemental Digital Content (Table S7, http://links.lww.com/JS9/E943, Tables S8, http://links.lww.com/JS9/E944, Table S9, http://links.lww.com/JS9/E945, Table S10, http://links.lww.com/JS9/E946, Table S11, http://links.lww.com/JS9/E947, Table S12, http://links.lww.com/JS9/E948) display the predictors for diagnoses in 2020 compared to 2019 for each of the six major cancers. For all six cancers, patients diagnosed in 2020 were more likely to be diagnosed with higher-grade cancers (grade I as the reference, at least one higher-grade subgroup showed OR > 1, *P* < 0.05). Additionally, for the four cancers other than lung and pancreatic cancer, patients diagnosed in 2020 were less likely to reside in areas with populations exceeding one million individuals (all OR < 1, *P* < 0.05).

### Impact of the COVID-19 pandemic on treatment and prognosis

Table 2 presents the time intervals from diagnosis to treatment. Compared to 2019, patients diagnosed in 2020 experienced shorter median time intervals from diagnosis to treatment for lung cancer (0.93 months vs. 1.00 months, *P* < 0.001), female breast cancer (1.07 months vs. 1.13 months, *P* < 0.001), prostate cancer (2.27 months vs. 2.37 months, *P* < 0.001), and corpus & uterus cancer (0.93 months vs. 1.03 months, *P* < 0.001). No significant differences were observed for colorectal cancer (0.57 months vs. 0.60 months, *P* = 0.056) and pancreatic cancer (0.67 months vs. 0.97 months, *P* = 0.070).

**Table 2:** Median time from diagnosis to treatment by year of diagnosis, 17 SEER registries, 2019–2020

Cancer sites	Year of diagnosis		*P*-value^b^
	2019^a^	2020^a^	
Lung & bronchus	1.00 (0.30–1.80)	0.93 (0.27–1.73)	<0.001
Female breast	1.13 (0.70–1.70)	1.07 (0.63–1.60)	<0.001
Prostate	2.37 (1.27–3.76)	2.27 (1.17–3.60)	<0.001
Colon & rectum	0.60 (0.00–1.27)	0.57 (0.00–1.20)	0.056
Pancreas	0.97 (0.60–1.50)	0.67 (0.60–1.33)	0.070
Corpus & uterus	1.03 (0.23–1.60)	0.93 (0.27–1.47)	<0.001

^a^ Time (months) is expressed as median (interquartile range, IQR).

^b^ Wilcoxon rank sum test.

**Figure 3.**
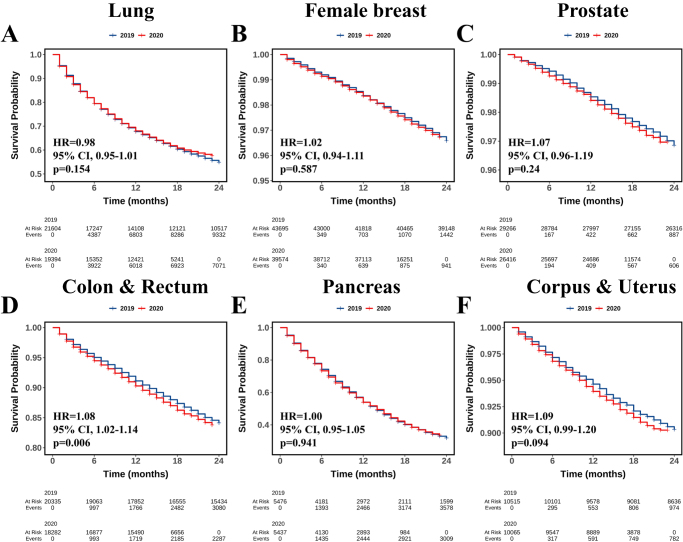
The Kaplan–Meier curves illustrate the cancer-specific survival for lung cancer (A), female breast cancer (B), prostate cancer (C), colorectal cancer (D), pancreas cancer (E), and corpus & uterus cancer (F) diagnosed between 2019 and 2020.

Figure 3 illustrates the impact of the COVID-19 pandemic in 2020 on CSS. Compared with patients diagnosed in 2019, those diagnosed in 2020 showed no significant differences in CSS for lung cancer (HR = 0.98, 95% CI, 0.95–1.01, *P* = 0.154, Figure 3A), female breast cancer (HR = 1.02, 95% CI, 0.94–1.11, *P* = 0.587, Figure 3B), prostate cancer (HR = 1.07, 95% CI, 0.96–1.19, *P* = 0.240, Figure 3C), pancreatic cancer (HR = 1.00, 95% CI, 0.95–1.05, *P* = 0.941, Figure 3E), and corpus & uterus cancer (HR = 1.09, 95% CI, 0.99–1.20, *P* = 0.094, Figure 3F). However, compared with patients diagnosed in 2019, patients diagnosed with colorectal cancer in 2020 exhibited significantly worse CSS (HR = 1.08, 95% CI, 1.02–1.14, *P* = 0.006, Figure 3D). Supplemental Digital Content Table S13 (http://links.lww.com/JS9/E949) details the 6-, 12-, and 18-month CSS rates for the six cancers.

## Discussion

Previous studies have demonstrated that the COVID-19 pandemic in 2020 has had multidimensional negative impacts on cancer management, including cancer screening, diagnosis, and treatment^[7–9]^. These adverse effects have been particularly pronounced in low- and middle-income countries as well as in nations that implemented lockdown measures^[9]^. Furthermore, patients with cancer have more commonly experienced economic hardships and deteriorations in psychosocial well-being during the pandemic^[21,22]^. Therefore, a comprehensive understanding of these impacts is critical for developing future health care policies and strengthening support systems to mitigate adverse effects in similar future crises.

Consistent with previous studies, our study observed a decline in the number of newly diagnosed cases of the six cancers in 2020 compared to 2019, with varying degrees of reduction^[9,23,24]^. One contributing factor may be the widespread reduction in cancer screening during the COVID-19 pandemic^[7,10]^. For instance, a meta-analysis by Teglia et al. reported that colorectal cancer screening rates decreased by 44.9% and female breast and cervical cancer screening rates decreased by 40% to 50% between January 2020 and October 2020^[10]^. Additionally, the pandemic may have led asymptomatic or mildly symptomatic patients to delay hospital visits, thereby reducing the number of new diagnoses and increasing the proportion of advanced-stage cases^[10,25]^.

As revealed by our stage-specific analysis, the decline in newly diagnosed distant-stage cancer cases was less marked compared to the reductions observed for localized and regional-stage cancers. Notably, cases of distant-stage pancreatic cancer and corpus and uterus cancer even showed an increase. These findings were further corroborated by our ordered logistic regression analyses, which demonstrated that, compared with 2019, five of the six cancers (excluding lung cancer) were diagnosed at more advanced stages in 2020. The lack of this phenomenon in lung cancer may be attributed to the partial overlap of its clinical symptoms with those of COVID-19, which likely increased clinical vigilance and facilitated earlier diagnosis. In fact, Thiruppathi et al. reported that lung cancer screening was not reduced during the 2020 pandemic period^[26]^.

Despite an overall reduction in the number of diagnoses during the COVID-19 pandemic, the extent appeared to vary across different subpopulations. In terms of cancer characteristics, in addition to the aforementioned effect on stage, another notable impact was observed in cancer grading.

During the COVID-19 pandemic in 2020, patients with all six types of cancer were more likely to be diagnosed with higher-grade tumors. This trend may be attributed to the fact that higher-grade cancers typically progress more rapidly and present with clinical symptoms earlier, making them less affected by reduced screening availability and longer delays in medical consultation. Another interesting observation was that, for four of the six cancers (excluding lung and pancreatic cancers), patients diagnosed in 2020 were less likely to reside in areas with populations over one million. This shift may be partly due to the reallocation of medical resources to address the surge in COVID-19 cases in large cities, leading to a reduction in routine services such as cancer screening and diagnosis. In addition, stricter lockdown measures and public health restrictions in densely populated areas may have further limited healthcare access and discouraged individuals from seeking medical care. These factors collectively reduced the usual advantages of early detection and screening in urban settings, highlighting the impact of healthcare accessibility and pandemic response strategies on cancer diagnosis patterns^[25]^.

Previous studies have widely reported delays in cancer treatment during the COVID-19 pandemic, with a more pronounced reduction in surgical treatments compared to drug therapies^[9,10,27,28]^. However, in our study, no delays in the time from diagnosis to treatment were observed for any of the six cancers; for four of them, the interval was even shortened. A previous study based on the SEER database has already noticed this phenomenon^[29]^. On the one hand, it might be due to an increased urgency for treatment resulting from a higher proportion of advanced-stage patients. On the other hand, the delays or reductions in treatment during the pandemic followed a U-shaped trend from January 2020 to October 2020, with the later months being less affected by the COVID-19 pandemic^[10]^. Our study, however, was unable to distinguish monthly variations, as it provided an average comparison that might not fully capture the most accurate temporal dynamics.

The multidimensional impact of the COVID-19 pandemic on cancer management has raised concerns about adverse impact on oncological outcomes. Hong et al. reported that the 1-year survival rate for patients with all cancer types decreased from 82.3% in the second quarter of 2018 to 77.5% during the second quarter of 2020^[16]^. In this study, a significant deterioration in CSS was observed among patients with colorectal cancer. In addition, similar trends were noted for prostate cancer and corpus & uterus cancer, although these did not reach statistical significance. Conversely, no obvious differences in prognosis were observed between patients diagnosed during and before the pandemic for lung cancer, female breast cancer, and pancreatic cancer. The potential explanations for these findings are multifactorial. On one hand, the pandemic may have exerted differential impacts on various cancer types. Moreover, the relatively short follow-up period may have limited our ability to detect significant differences, highlighting the need for long-term follow-up to elucidate the pandemic’s impact on survival.

## Limitations

Several limitations of this study need to be acknowledged. First, its retrospective and cross-sectional design introduces inherent biases and limits causal inference. Second, the follow-up time for patients diagnosed in 2020 was relatively short, with a maximum of approximately 18 months, which may underestimate the long-term impact of the COVID-19 pandemic on survival. Third, the inability to perform month-by-month analyses may have obscured important temporal variations and pandemic-related disruptions during different stages of 2020. Fourth, the study focused only on six types of cancer and specific geographic coverage, which may limit the generalizability of the findings to other populations, cancer types, or healthcare systems. Lastly, unmeasured confounding factors, such as shifts in healthcare policies, resource allocation, and individual health-seeking behaviors during the pandemic, may have influenced the associations observed and were not fully adjusted for.

## Conclusion

The number of diagnosed cases for the six major cancers decreased during the COVID-19 pandemic in 2020, but the impact varied across different population characteristics. In 2020, most cancer types were diagnosed at more advanced stages, yet the timeliness of treatment was not adversely affected. Some cancer types exhibited significantly worse CSS or trends, but longer follow-up is needed to confirm these findings.

## Data Availability

Data is available from the corresponding author if justification for the requirement is justified

## Research registration unique identifying number (UIN)

This study is recorded in the Research Registry with a unique registration ID of researchregistry11152.
